# Quality of life, food tolerance, and eating disorder behavior after laparoscopic gastric banding and sleeve gastrectomy - results from a middle eastern center of excellence

**DOI:** 10.1186/s40608-018-0220-6

**Published:** 2018-12-27

**Authors:** Khalid Al Khalifa, Ahmed Al Ansari

**Affiliations:** 1 Department of General Surgery, Bahrain Defence Force Hospital, P. O. Box - 28743, Riffa, Bahrain; 2 Training and Education Department, Bahrain Defence Force Hospital, Off Waly Alahed Avenue, West Riffa, P. O. Box - 28743, Riffa, Bahrain

**Keywords:** Bariatric surgery, Gastric sleeve, Gastric band, Quality of life, Food tolerance, Eating disorders

## Abstract

**Background:**

Obesity is a major health problem in Arab countries. Bariatric surgery can improve the quality of life of an obese individual. However, different types of bariatric surgery result in varying levels of food intolerance as a side effect. Many patients who undergo bariatric surgery are also at risk of subsequently developing eating disorder behaviors. The aim of the study was to compare the quality of life, food tolerance, and behaviors of eating disorders related to laparoscopic sleeve gastrectomy and gastric banding.

**Methods:**

A retrospective review of medical records and a questionnaire-based survey was completed for all patients who had undergone either bariatric sleeve gastrectomy or gastric banding at the Bahrain Defense Force Hospital between 2011 and 2014. Each patient was administered 3 questionnaires to assess the quality of life, food tolerance, and eating disorder behaviors.

**Results:**

Forty-eight patients who had undergone sleeve gastrectomy and 36 who had undergone gastric banding participated in the study. Sleeve gastrectomy patients showed better food tolerance (*P* < 0.001) and better eating behaviors (*P* = 0.001) post-surgery compared with gastric banding patients. Health-related quality of life (HRQOL) did not differ significantly between the 2 groups. Only sleeve patients had preoperative evaluation of these parameters (HRQOL). However, in the gastric sleeve group, after the surgery, significant improvement was found in all parameters of HRQOL except for mental health status.

**Conclusion:**

Laparoscopic gastric sleeve surgery patients had superior outcomes in both food tolerance and eating disorder behaviors. The quality of life did not significantly differ between the gastric sleeve and gastric banding surgery groups.

## Background

According to the World Health Organization, the prevalence of overweight and obesity in Eastern Mediterranean countries is 74 to 86% in women and 69 to 77% in men [1]. The prevalence of obesity in adults ranges from 1 to 30% among males and 2 to 55% among females [2]. Bariatric surgery has been established as a safe and effective weight loss method in obese patients. It has become popular worldwide, with the number of procedures performed annually tripling between 2003 (143,301 procedures) and 2013 (468,609 procedures) [3]. Although both laparoscopic sleeve gastrectomy (LSG) [4] and laparoscopic adjustable gastric banding (LAGB) [5] cause satisfactory weight loss, more effective weight loss is seen with sleeve gastrectomy than with gastric banding [6].

A patient’s quality of life markedly improves early within several months after bariatric surgery [7], with a persistent effect seen at 2-year [8] and 10-year follow-ups [9]. A higher body mass index (BMI) is strongly associated with a poorer quality of life [10], with the effects being stronger among females [11]. Other than weight loss, health-related quality of life (HRQOL), food tolerance, and behaviors of eating disorders are also important parameters that determine the success of bariatric surgery. The assessments of personality, serious psychiatric ailments at baseline, and the recuperation of depressive symptoms are also a part of the postoperative HRQOL assessment. The postoperative continuation of psychiatric symptoms and incorrect eating behaviors contribute to poor HRQOL outcomes as well [12].

In a review of 15 research studies, 14 studies reported that binge eating (BE), BE disorder (BED), and loss of control (LOC) eating are common behaviors after bariatric surgery and are associated with poorer weight loss outcomes and/or more weight gain after the surgery [13]. Clearly, eating disorder (ED) symptoms are present in a substantial number of post-bariatric surgery patients [14]. These cases are, however, not reported due to their sub-syndromal appearance, which—to our knowledge—has not been studied in the Middle East. After undergoing bariatric surgery, patients may present with recurrent vomiting, malabsorption, poor postoperative nutrient intake, and poor compliance with vitamin supplement intake and regular follow-up [15]. Reduced food intake, inferior dietary quality, changes in digestion and absorption, and non-adherence to supplementation regimens often lead to the deficiency of micronutrients, such as thiamine, vitamin B12, vitamin D, iron, and copper [16]. Food tolerance and gastrointestinal quality of life are much better after sleeve gastrectomy than after gastric banding, 2 to 4 years post surgery [17].

We examined and compared the quality of life, quality of alimentation, and behaviors of eating disorders associated with LSG and gastric banding in patients treated at our military training hospital. To our knowledge, this is the first study to examine such issues in the Arabian Gulf region, and hence, we believe that it will provide further insight for future research on bariatric surgery trends in the region.

## Methods

The purpose of the study was to perform a comparison about how the quality of life, food tolerance and eating disorder behavior varied according to different bariatric surgeries such as gastric sleeve and gastric band surgeries. After approval was obtained from the institutional research and ethics committee, a retrospective chart review was completed for all patients who had undergone laparoscopic gastric banding and sleeve gastrectomy at the Bahrain Defense Force Hospital between 2011 and 2014. In 2015, we telephoned all patients who had at least 1 year of postoperative duration and invited them to join the follow-up survey. Those who responded to our phone call were invited to participate in the follow-up questionnaire survey at the hospital. We scheduled the survey on 2 different days so that the patients could come and complete the survey on any of those 2 days according to their convenience. All patients were assessed through face-to-face interviews with a dietitian. We included gastric sleeve participants those that had both pre and post-operative QOL evaluations. As the gastric band patients preoperative assessments were not available for comparison, we included all the eligible gastric band patients for the post-operative comparison in the study.

A total of 84 bariatric patients, each of whom had undergone a gastric sleeve or gastric band procedure that a single surgeon conducted, were included in the study. Of these, 48 were gastric sleeve patients, and 36 were gastric band patients. After they completed psychiatric evaluations, we sent them for further clinical assessment. The changes in BMI were evaluated and compared between the surgical groups by using the Mann-Whitney U test. Patients were asked to complete 3 questionnaires: the Medical Outcomes Study Short-Form Questionnaire (SF-36), the Quality of Alimentation questionnaire, and the Eating Disorder Examination Questionnaire (version 6; EDE-Q).

The SF-36 is a 36-item questionnaire that evaluates eight different aspects of quality of life: physical functioning, physical role functioning, bodily pain, general health, vitality, social functioning, emotional role functioning, and mental health [11]. The responses to this self-reported questionnaire were eventually grouped into a physical health category and a mental health category.

The Quality of Alimentation questionnaire [18] was developed to assess food tolerance after bariatric surgery. It consists of 4 components, including overall patient satisfaction with alimentation, the timing and content of meals and snacks, the tolerance of different types of foods, and the frequency of vomiting. A Mann-Whitney U test was conducted to assess the food tolerance between the 2 groups.

The EDE-Q [19] was used to assess eating-disorder psychopathology in a self-reported manner, such that patients described their eating behaviors during a 28-day period immediately before the administration of the questionnaire. This questionnaire was distributed to the patients before they were interviewed individually. The questions asked were related to the following 4 categories: restraint, eating concerns, shape concerns, and weight concerns. This questionnaire differentiates objective bulimic episodes from subjective bulimic episodes and objective overeating.

The Statistical Package for Social Science (SPSS Version 19.0) was used to analyze the data. The results are presented as a mean ± standard deviation (SD) or percentage of patients. The pre- and postoperative comparisons were calculated to evaluate the changes of the quality of life of gastric sleeve patients. The Shapiro-Wilk test was used for testing the normality. The data did not follow the parametric assumptions but were positively skewed. Thus, we used non-parametric tests. Both groups were compared using the Mann-Whitney U test. To assess the differences in the participants’ quality of life before and after their gastric sleeve procedures, the Wilcoxon Signed-Rank Test was used. Qualitative data were compared using the chi-square test or Fisher’s exact test as appropriate. The correlation between excess weight loss (EWL) and postoperative quality of life, food tolerance, and eating disorder scores were assessed by using the Spearman rank correlation coefficient. The percent of excess weight loss (%EWL) was calculated as follows: [(operative weight–follow-up weight) / (operative weight–ideal weight)] × 100, with the ideal weight being based on a BMI of 25 kg/m^2^. A *P* value of < 0.05 was considered significant.

## Results

### Demographics

The mean age of the patients was 35.8 ± 10.1 years in the gastric sleeve group and 36.1 ± 10.3 years in the gastric band group. The proportion of women was higher (58.3%). No differences in age (*P* = 0.681) or gender distribution (*P* > 0.05) were observed between the groups. The follow-up rate was 100% for gastric banding, and 92% for sleeve gastrectomy. The follow-up period ranged from 1 to 4 years and the median duration of follow-up was two years for gastric sleeve patients. However, the median follow-up time for gastric band patients was 3 years with follow-up period ranged from 1 to 4 years. The mean BMI dropped from 48.2 ± 9.6 kg/m^2^ to 30.9 ± 6.2 kg/m^2^ after surgery in the gastric sleeve group, and from 44.2 ± 4.4 kg/m^2^ to 34.9 ± 6.8 kg/m^2^ in the gastric band group. The preoperative BMI did not significantly differ between the groups (48.2 ± 9.6 vs. 44.2 ± 4.5*, P* = 0.107). However, the postoperative BMI reduction was greater in the gastric sleeve group (31.0 ± 6.2) than in the gastric band group (34.9 ± 6.8), *P* = 0.012. The %EWL was significantly higher in the sleeve gastrectomy group (75.3 ± 23.9) than the gastric band group (48.4 ± 3.0), *P* = 0.001.

### SF-36 questionnaire

The analysis of the SF-36 questionnaire responses indicated that postoperative quality of life did not significantly differ between the groups. Assessments of the pre-and postoperative HRQOL of the patients in the gastric sleeve group exhibited significant improvements in all of the items except for mental health domain. (Table 1) However, there was no pre-operative HRQOL data available for gastric banding patients.

**Table 1:** HRQOL by surgery group assessed by the SF-36

Subscale category	Gastric Sleeve (*n* = 48)			Gastric Band (*n* = 36)	*P* value^2^
	Preoperative	Postoperative	*P* value^1^	Post-operative	
Physical functioning	54.4 ± 30.6	85.0 ± 22.9	< 0.001*	91.0 ± 33.7	0.677
Physical role limitation	52.1 ± 43.1	85.2 ± 30.6	< 0.001*	85.4 ± 31.8	0.946
Body pain	64.1 ± 30.6	80.1 ± 29.1	0.025*	76.04 ± 31.4	0.368
General health	62.0 ± 19.9	76.2 ± 22.0	0.002*	70.08 ± 22.7	0.188
Vitality	52.6 ± 16.8	68.2 ± 22.4	< 0.001*	60.00 ± 21.0	0.106
Social functioning	68.2 ± 27.8	84.2 ± 26.1	0.008*	83.7 ± 22.9	0.47
Emotional role limitation	62.5 ± 40.5	87.1 ± 28.7	0.001*	80.6 ± 34.2	0.279
Mental health	66.8 ± 18.8	69.6 ± 21.8	0.566	63.7 ± 21.3	0.206

*P* value of < 0.05 was considered significant. Data expressed as mean ± SD

*P* value^1^: Wilcoxon signed rank test, comparison between pre and post-operative gastric sleeve patients’ quality of life.; *P* value ^2^: Mann-Whitney U test, comparison between Post –operative quality of life between Gastric sleeve and Band

*P < 0.05

### Quality of alimentation questionnaire

The postoperative overall food tolerance and the evaluation of different food scores of the gastric sleeve group and gastric band group were significantly different (*P* = 0.003 and *P* < 0.001 respectively). Moreover, the frequency of vomiting was greater in the gastric sleeve group than in the gastric band group (P < 0.001), and the total score was significantly higher in the gastric sleeve group (*p* < 0.001). The findings of the Quality of Alimentation questionnaire are shown in Table 2.

**Table 2:** Food tolerance measured using the Quality of Alimentation questionnaire

	Gastric banding (*n* = 36)	Gastric sleeve surgery (*n* = 48)	*P* value
Overall satisfaction with food intake	3.2 ± 1.0	3.9 ± 1.1	0.003*
Evaluation of different foods	8.8 ± 3.2	12.0 ± 3.4	< 0.001*
Frequency of vomiting	1.6 ± 1.9	4.5 ± 1.7	< 0.001*
Total score	13.6 ± 5.0	20.1 ± 5.0	< 0.001*

^*^P value of < 0.05 was considered significant. Data are expressed as mean ± SD. The composite score ranged from 1 to 27. Score 27 for being the maximum for an excellent food tolerance

*p* value from Mann-Whitney U test

### Ede-q

The mean global EDE-Q scores were significantly different between the groups (2.4 ± 1.2 vs. 3.2 ± 0.9; *P* = 0.001). However, the restraint subscale did not significantly differ between the groups (1.5 ± 1.6 vs. 1.8 ± 1.5; *P* = 0.296). Patients from the gastric band group showed a significantly greater number of eating disorder behavior traits as compared with those from the gastric sleeve group (Table 3).

**Table 3:** Postoperative behaviors of eating disorders of patients, as assessed by the EDE-Q

Subscale category	Gastric sleeve surgery (*n* = 48)	Gastric banding (*n* = 36)	*P* value
Global EDE score	2.4 ± 1.2	3.2 ± 0.9	0.001*
Restraint	1.5 ± 1.6	1.8 ± 1.5	0.296
Eating concern	1.7 ± 1.3	2.6 ± 1.3	0.006*
Shape concern	3.3 ± 1.5	4.5 ± 1.2	< 0.001*
Weight concern	3.1 ± 1.4	4.0 ± 1.2	0.001*

^*^P value of < 0.05 was considered significant. Data expressed as mean ± SD

*p* value from Mann-Whitney U test. Higher scores indicate greater levels of symptomatology

A Spearman rank correlation test was conducted to analyze the positive changes in the quality of life after surgery, as well as the changes in weight loss (%EWL). We observed that quality of life and weight loss were positively and significantly correlated (*r* = 0.478, *P* = 0.003) in the gastric band group, but not significantly correlated in the gastric sleeve group. However, food tolerance and weight loss were not significantly correlated in either group.

In addition, the postoperative behaviors of eating disorders were found to be significantly and negatively correlated (*r* = − 0.367, *P* = 0.010) with weight loss in the gastric sleeve group.

## Discussion

As the number of obese individuals continues to increase worldwide, obesity is expected to remain a challenging health issue [20]. Bariatric surgery has proved to be the most effective method for maintaining weight loss among morbidly obese individuals [21].

Gastric sleeve surgery is considered easier to perform than other bariatric surgeries, as the pylorus is preserved [22]. Yet, both gastric sleeve surgery and gastric band surgery result in marked improvement in quality of life with no significant difference between the 2 surgeries [23]. In France, researchers used the SF-36 questionnaire and found significant improvement in all domains of quality of life at 1 year after gastric sleeve surgery [9]. In a study similar to ours, a Polish version of the SF-36 questionnaire as well as the Moorhead-Ardelt quality of life assessment tool was used. The researcher noted that both gastric sleeve and laparoscopic Roux-en-Y surgeries were associated with improved quality of life with no significant difference between the procedures [23]. These findings are consistent with those of our comparison of gastric sleeve surgery and gastric band surgery. Strain et al. observed that all patients had poor quality of life at baseline before bariatric surgery (as assessed by the SF-36); however, the scores of all of the patients improved after surgery, regardless of the type of surgery [24]. We found similar results. The post-operative quality of life improvements did not show any significant difference between the gastric sleeve and gastric banding groups.

After bariatric surgery, many people develop food intolerance, reportedly due to the development of an aversion to certain types of foods, which may translate into vomiting or dumping syndrome [25]. Sleeve gastrectomy procedures are associated with the least number of food intolerances and generally do not cause dumping syndrome [25]. In addition, in our study, sleeve gastrectomy patients experienced fewer food intolerances compared with gastric banding patients.

Vomiting is the most common food intolerance complaint among patients who have undergone gastric banding surgery [14]. In our study, a greater number of vomiting incidents were noted in gastric sleeve surgery patients than in gastric banding patients. One explanation for this discrepancy may be that the patients who experienced vomiting were not closely adhering to the dietary recommendations. Gastric banding patients reported an overall lower level of food tolerance. This is consistent with other studies [26].

Disordered eating behaviors, including binging, grazing, and stress eating, are correlated with poorer weight loss outcomes at 1 year post surgery [27]. In our study, gastric sleeve patients who showed fewer disordered eating behaviors after surgery had the best weight loss outcomes. Grazing has been reported in 19.5–59.8% patients after bariatric surgery [14]. Eating disorder traits that are present in patients preoperatively are significantly reduced after bariatric surgery [28]^;^ this trend was also observed in our study.

### Limitations

This study was limited by the small sample size. Although the study showed a clear trend in weight loss and improved quality of life, a larger sample size may have provided deeper insights into the topic and may have enabled a wider extrapolation of the results. Another limitation is that this study does not include pre-operative HRQOL data for gastric banding patients. It is important to have pre-operative HRQOL data for patients undergoing each surgery to ensure whether there were significant differences in HRQOL after each surgery. Therefore, the lack of pre-operative data makes it difficult to suggest that the differences in HRQOL results were due to the different types of surgery performed. Future studies should collect pre-operative data in order to demonstrate that the differences in HRQOL were only significant post-operatively, this would allow the study to suggest the possibility of the differences being due to the different kinds of surgery performed. Additionally although this study provides results from patients in the Arabian gulf region, it has only been conducted in one country. Therefore, more studies are required in order for it to be generalized to the Arabian gulf region and other populations.

## Conclusion

Quality of life did not significantly differ between the 2 groups. Both surgeries led to improvements in almost all of the items tested by using the 3 questionnaires. To our knowledge, no study in the Middle East has examined quality of life, food tolerance, and behaviors of eating disorders between patients undergoing sleeve gastrectomy and gastric banding. Further studies are needed to compare sleeve gastrectomies with other bariatric surgeries to evaluate whether it is indeed a better procedure for obese patients. Dysfunctional eating behaviors after laparoscopic gastric banding and sleeve gastrectomy appear to be associated with poorer weight loss and/or weight regain. The early diagnosis of such behaviors can help to prevent poor outcomes of the surgeries.

## Abbreviations

%EWL: % Excess Weight Loss
BMI: Body Mass Index
EDE-Q: Eating Disorder Examination Questionnaire
HRQOL: Health Related Quality Of Life
LAGB: Laparoscopic Adjustable Gastric Banding
LSG: Laparoscopic Sleeve Gastrectomy
SF-36: Short-Form Questionnaire
